# Bilateral Asymmetries of Plantar Pressure and Foot Balance During Walking, Running, and Turning Gait in Typically Developing Children

**DOI:** 10.3390/bioengineering12020151

**Published:** 2025-02-05

**Authors:** Wei Liu, Liu Xu, Haidan Wu, Yile Wang, Hanhui Jiang, Zixiang Gao, Endre Jánosi, Gusztav Fekete, Qichang Mei, Yaodong Gu

**Affiliations:** 1 Faculty of Sports Science, Ningbo University, Ningbo 315211, Chinagaozixiang0111@outlook.com (Z.G.);; 2 Faculty of Engineering, University of Pannonia, 8201 Veszprem, Hungary; 3 Savaria Institute of Technology, Faculty of Informatics, Eötvös Loránd University, 9700 Szombathely, Hungary; je@inf.elte.hu; 4 Department of Material Science and Technology, AUDI Hungária Faculty of Vehicle Engineering, Széchenyi István University, 9026 Győr, Hungary; 5 Auckland Bioengineering Institute, The University of Auckland, Auckland 1010, New Zealand

**Keywords:** children gait, plantar pressure distribution, foot balance, center of pressure, movement asymmetry, SPM1D

## Abstract

Biomechanical asymmetries between children’s left and right feet can affect stability and coordination, especially during dynamic movements. This study aimed to examine plantar pressure distribution, foot balance, and center of pressure (COP) trajectories in children during walking, running, and turning activities to understand how different movements influence these asymmetries. Fifteen children participated in the study, using a FootScan plantar pressure plate to capture detailed pressure and balance data. The parameters, including time-varying forces, COP, and Foot Balance Index (FBI), were analyzed through a one-dimensional Statistical Parametric Mapping (SPM1d) package. Results showed that asymmetries in COP and FBI became more pronounced, particularly during the tasks of running and directional turns. Regional plantar pressure analysis also revealed a more significant load on specific foot areas during these dynamic movements, indicating an increased reliance on one foot for stability and control. These findings suggest that early identification of asymmetrical loading patterns may be vital in promoting a balanced gait and preventing potential foot health issues in children. This study contributes to understanding pediatric foot biomechanics and provides insights for developing targeted interventions to support healthy physical development in children.

## 1. Introduction

The human foot has 28 bones, 33 joints, 112 ligaments, 13 extrinsic muscles, and 21 intrinsic muscles [1]. The foot is one of the most significant bodily elements and an essential component of locomotion [2]. The muscles, ligaments, and tendons associated with the foot bones are essential in maintaining overall form and ensuring function under static or dynamic conditions [3]. Differences in foot structure are associated with differences in foot function during static postures or dynamic movements. Many pathologies of the foot have a biomechanical origin and are usually related to foot type [4,5,6].

Intrinsic and extrinsic foot muscles control the arch [7] to respond to the increased load transport and running demands [8], suggesting that the arch is the “core” of the foot and is essential to normal foot function [9]. The foot of children develops quickly between the ages of 7 and 12 [9], and it has been reported that male arch height increases between ages 6 and 13 and female arch increases between ages 8 and 13 [10]. Previous findings demonstrated that child foot size and bone structure throughout average childhood growth are impacted by variations in gender and age. According to this biological nature, it has been shown that children’s feet usually have a neutral and internally rotated foot posture, which frequently leads to aberrant foot morphology [11], thus affecting the daily location.

During walking and running, the arches of different morphologies and structures also cause changes in plantar pressure and load [12,13,14,15]. A study on preschool children found that the mean forces and pressures beneath the first and second metatarsals and the midfoot during the stance phase of walking were associated with the navicular heights and foot arch volumes [12]. Children’s shoes with arch support are a non-surgical corrective method to affect the changes in plantar pressure caused by foot shape differences [11,13]. With the arch support structure, the average plantar contact area of the midfoot increased during running [13]. A recent study investigating gait turning with different angles [16], reporting that plantar pressure patterns shifted during the beginning of the approaching step. However, there are only a few studies focusing on biomechanics and plantar loadings while executing the walking, running, and turning tasks.

Although there has been much research on foot shape changes and growth patterns in children, there has yet to be research on the symmetry/asymmetry in plantar loading profiles between the right and left foot in children. Therefore, the purpose of this study was to explore the loading and COP differences between the left and right feet of children in completing four maneuvers: walking, walking turn, running, and running turn.

## 2. Materials and Methods

### 2.1. Participants

Fifteen healthy female children (average age: 7.0 ± 1.3 years; height: 128.4 ± 6.9 cm; weight: 23.2 ± 3.8 kg) participated in this study. All participants were free of lower-limb injuries or surgeries within the past six months. Each participant’s dominant leg was identified based on their preferred kicking foot [17], with all participants being right-leg dominant. Before the study, both participants and their guardians received a thorough explanation of the procedures and provided informed consent. Ethical approval was obtained from the university’s research ethics committee.

### 2.2. Test Protocol

**Figure 1:**
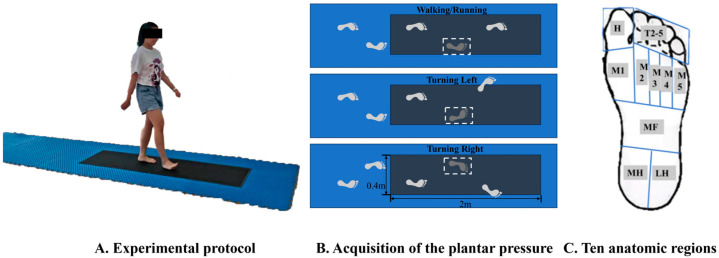
Illustration of the test protocol of straight running (**A**), left and right turning (**B**), and division of anatomic regions (**C**).

Data were collected on a FootScan plantar pressure plate (RsScan International, Olen, Belgium) embedded in a 20-m walkway. The plate, measuring 2 m × 0.4 m × 0.02 m with a default sampling frequency of 480 Hz, was calibrated for each participant’s weight before testing [18]. Each participant performed four tasks in a barefoot condition: straight walking, straight running, turning during walking (left and right), and turning during running (left and right). For turning tasks, the stance leg was analyzed (e.g., right foot for left turns, left foot for right turns) (Figure 1A,B). Participants performed a minimum of three successful trials per task, and any trials with incomplete steps on the pressure plate were excluded. Foot pressure data were collected from ten anatomical regions (Figure 1C), including the big toe (Toe 1), other toes (T2–5), metatarsals I–V (M1–M5), midfoot (MF), medial heel (MH), and lateral heel (LH).

### 2.3. Data Processing

Time series parameters for each stance phase were interpolated to 101 points using a cubic spline to ensure uniformity and represent 100% of the stance [19]. The Foot Balance Index (FBI), calculated from the average peak pressures of metatarsal and heel regions, indicated foot stability, with positive values denoting pronation and negative values denoting supination. The trajectory of the Center of Pressure (COP) during each stance phase was also interpolated to 101 data points for statistical comparison.

The Foot Balance Index (FBI) was calculated using the mean peak pressure values from the metatarsal and heel regions, as shown in Equation (1). M1, M2, M3, M4, and M5 represent the five metatarsal regions, and MH and LH represent the Medial Heel (MH) and Lateral Heel (LH) regions. *Favg* means average force over the stance. The FBI provides a measure of overall foot stability:, where a positive value indicates pronation and a negative value indicates supination [18,20]:

(1) $$FBI=\frac{\left(M1+M2+MH\right)-(M3+M4+M5+LH)}{Favg}\times 100\%$$

The Center of Pressure (COP) trajectory, a key indicator of gait characteristics, was analyzed for each stance phase across walking, running, and turning tasks [21,22,23]. Both the COP and FBI trajectories were interpolated to a standard length of 101 data points using a cubic spline method, ensuring consistency for statistical analysis.

### 2.4. Statistical Analysis

To capture consistent performance, three trials for each movement task—walking, running, turning while walking, and turning while running—were averaged per participant to reduce trial variability. Force data were then normalized by *Z_avg_*, derived by dividing the total force by the sum of the original data frames [24,25,26]. Time-series data, including force, Center of Pressure (COP), and Foot Balance Index (FBI), were checked for normality before analysis. Statistical comparisons were conducted using the Statistical Parametric Mapping (SPM1d) method with independent sample t-tests based on random field theory [27,28]. All analyses were performed in MATLAB R2018a (The MathWorks, Natick, MA, USA), with results presented as mean values and standard deviations (SD). A two-tailed *p*-value of less than 0.05 was set as the threshold for statistical significance.

## 3. Results

### 3.1. Center of Pressure Trajectory

**Figure 2:**
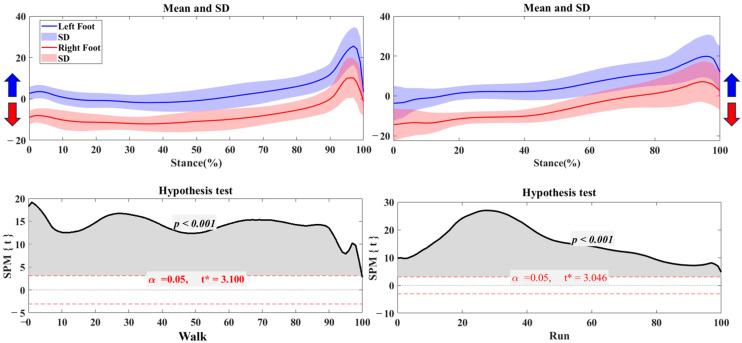
The COP of trajectory in the left and right foot during walking and running with the highlighted direction of pronation (Blue arrow) and supination (Red arrow). Note: Y-axis (%foot width).

Figure 2 illustrates the differences in the center of pressure (COP) trajectory in the left foot and right foot during both walking and running. During the stance phase, the left foot’s COP shifts toward pronation, while the right foot’s COP shifts toward supination. Hypothesis testing reveals significant differences in COP distribution throughout the stance phase for walking and running (*p* < 0.001).

**Figure 3:**
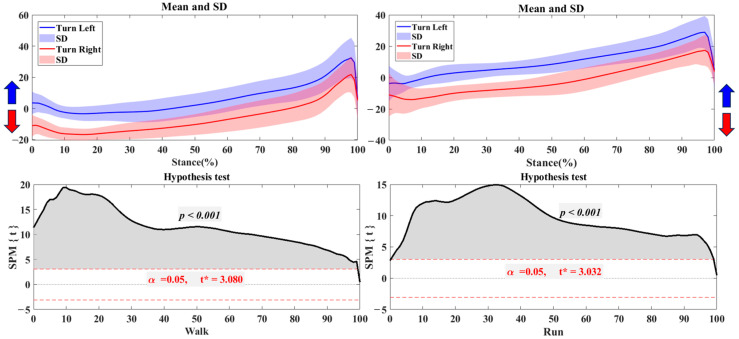
The COP of trajectory in the turning right/left during walking and running with the highlighted direction of pronation (Blue arrow) and supination (Red arrow). Note: Y-axis (%foot width).

Figure 3 illustrates the differences in the center of pressure (COP) trajectory when turning left versus right during walking and running. During the stance phase of children’s gait, the COP shifts pronation when turning left and toward supination when turning right. The standard deviation highlights that this trend is especially noticeable in running conditions. The hypothesis test results confirm significant differences in COP distribution throughout the stance phase of children’s gait for both walking and running (*p* < 0.001).

### 3.2. Foot Balance Index

**Figure 4:**
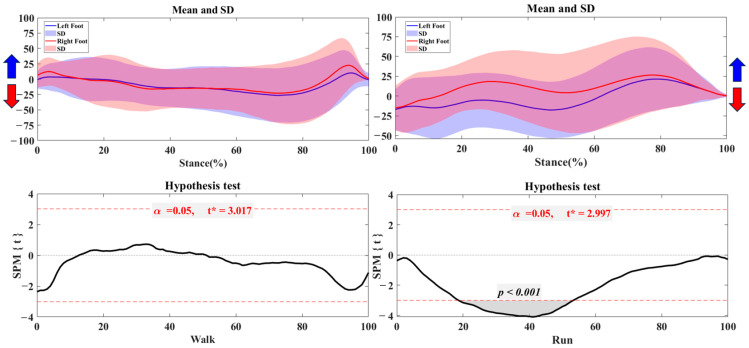
The FBI of trajectory in the left and right foot during walking and running with the highlighted direction of pronation (Blue arrow) and supination (Red arrow). Note: Y-axis (%).

Figure 4 shows that there are distinct differences in the Foot Balance Index (FBI) between the left and right feet during both walking and running. The left foot tends toward pronation (negative FBI values), while the right foot shows a tendency for supination (positive FBI values). This imbalance is more pronounced during running, with wider variations in the FBI, particularly for the right foot. Statistical tests confirm these differences, showing a highly significant result (*p* < 0.001) during the 18%–53% phase of running, indicating an increased asymmetry in foot balance in this condition.

**Figure 5:**
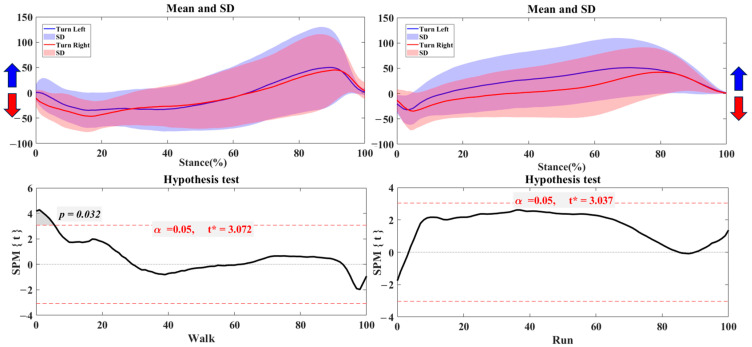
The FBI of trajectory in the turning right/left during walking and running with the highlighted direction of pronation (Blue arrow) and supination (Red arrow). Note: Y-axis (%).

Figure 5 shows the Foot Balance Index (FBI) results for left and right turns during walking and running. During left turns, the foot shifts towards pronation, while right turns shift towards supination. This pattern is more pronounced during running, particularly in the mid-stance phase, where deviations and variability are greater. However, no statistically significant differences between left and right turns in walking or running were found.

### 3.3. Regional Plantar Forces

**Figure 6:**
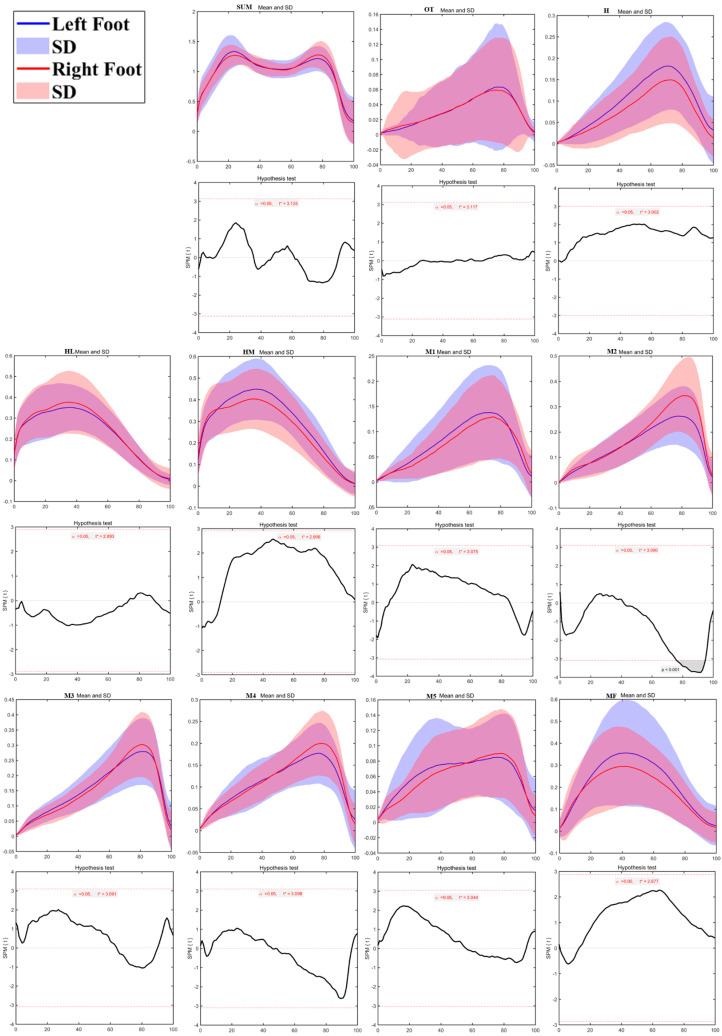
The sum of plantar pressure (SUM) and regional plantar forces during walking on the left foot and right foot with highlighted statistics. Note: Y-axis (normalized using Zavg).

As shown in Figure 6, plantar pressure distribution testing revealed significant differences between the left and right sides during walking. The M2 region on the left side had notably lower pressure than the right side during 76%–95% of the contact phase (*p* < 0.01). No significant pressure differences were observed in other plantar regions. 

**Figure 7:**
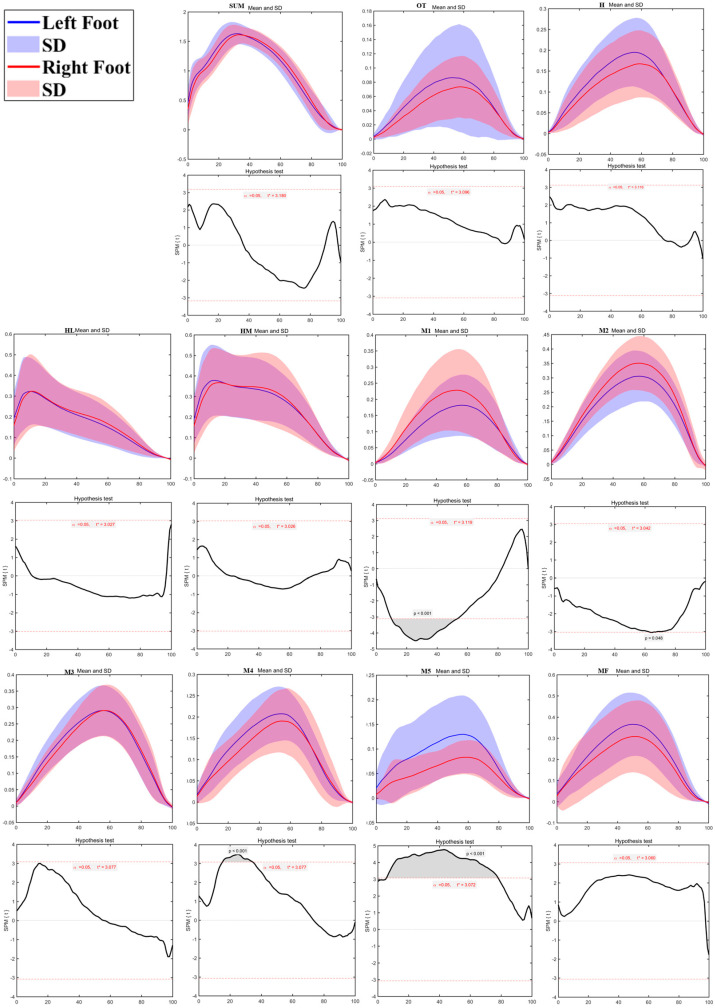
The sum of plantar pressure (SUM) and regional plantar forces during running in the left foot and right foot with highlighted statistics. Note: Y-axis (normalized using Zavg).

As shown in Figure 7, plantar pressure distribution testing revealed significant differences between the left and right sides during running. The M1 region on the left side showed significantly lower pressure than the right side during 10%–54% of the contact phase (*p* < 0.01). In contrast, the M4 region on the left side exhibited significantly higher pressure than the right side from 16%–35% (*p* < 0.01), and the M5 region showed significantly higher pressure from 5%–78% of the contact phase (*p* < 0.01). No significant differences were found in the other plantar regions.

**Figure 8:**
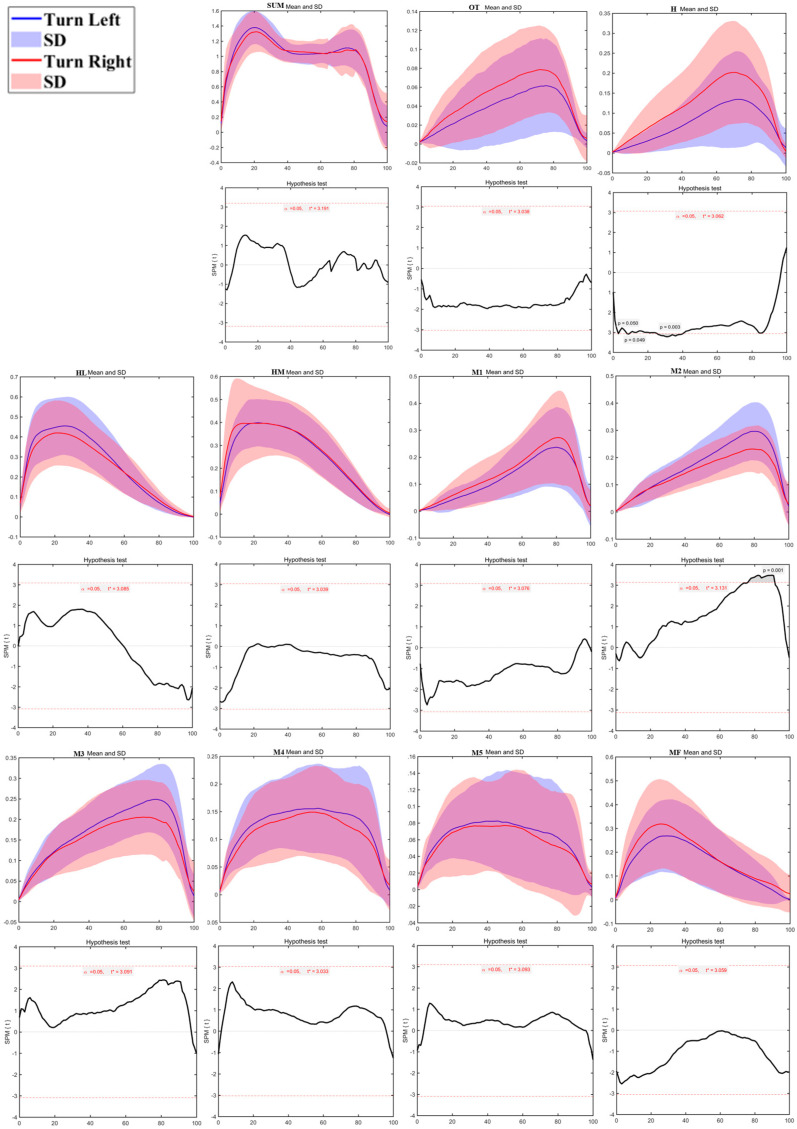
The sum of plantar pressure (SUM) and regional plantar forces during Turning Walking Tasks in the left turn and right turn with highlighted statistics. Note: Y-axis (normalized using Zavg).

As shown in Figure 8, plantar pressure distribution testing revealed significant differences between the left and right sides during walking turns. The H region during the Turn Left task showed significantly lower pressure compared to the Turn Right task at 3%–4% (*p* = 0.05), 8%–9% (*p* = 0.49), and 25%–38% of the contact phase (*p* < 0.01). In contrast, the M2 region during the Turn Left task exhibited significantly higher pressure than the Turn Right task from 76%–92% of the contact phase (*p* < 0.01). No significant differences were found in the other plantar regions.

**Figure 9:**
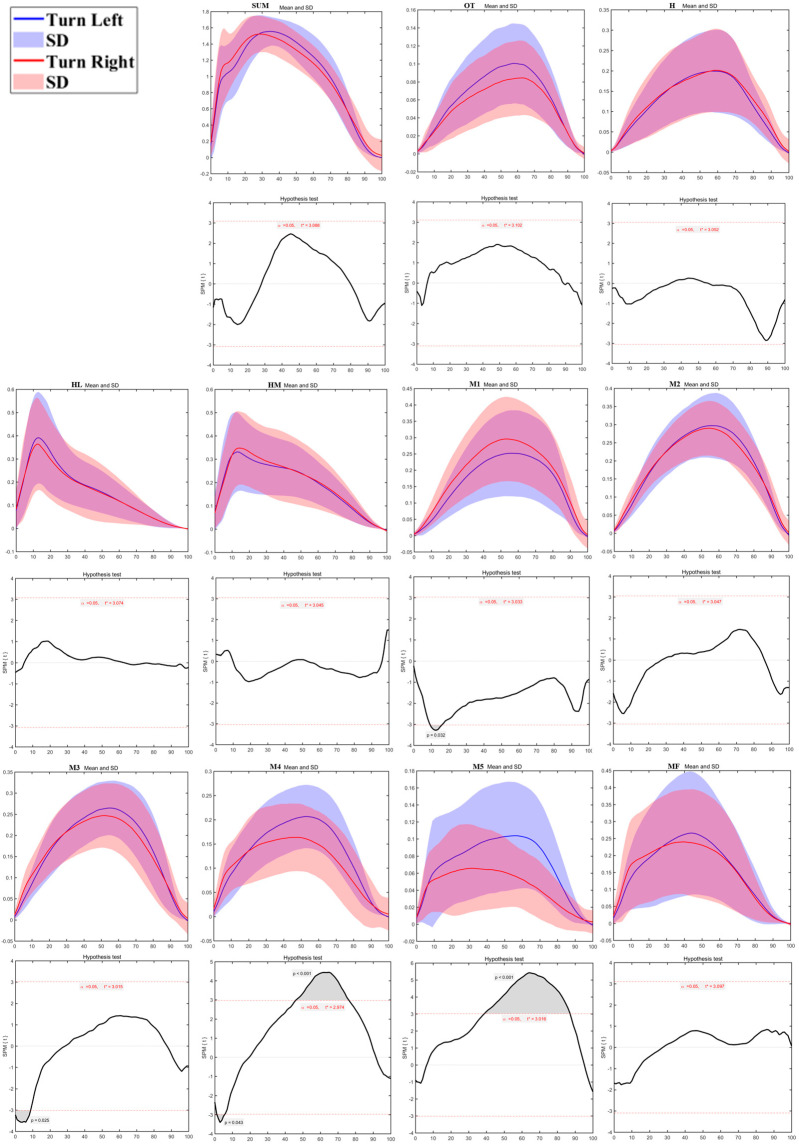
The sum of plantar pressure (SUM) and regional plantar forces during Turning running Tasks in the left turn and right turn with highlighted statistics. Note: Y-axis (normalized using Zavg).

As shown in Figure 9, plantar pressure distribution testing revealed significant differences between the left and right sides during the running turn. The M1 region during the True Left task showed significantly higher pressure than the True Right task from 9%–16% of the contact phase (*p* = 0.032). The M4 region exhibited significantly higher pressure from 46%–76% (*p* < 0.01), and the M5 region showed significantly higher pressure from 39%–88% of the contact phase (*p* < 0.01). Conversely, the M3 region during the True Left task demonstrated significantly lower pressure than the True Right task from 0%–9% (*p* = 0.025), and the M4 region showed lower pressure from 1%–5% of the stance phase (*p* = 0.043). No significant differences were found in the other plantar areas.

## 4. Discussion

This study provides an in-depth examination of biomechanical differences between children’s left and right feet across multiple movement tasks, specifically walking, running, walking turns, and running turns. Using metrics such as the center of pressure (COP) trajectory, Foot Balance Index (FBI), and regional plantar pressure, we identified that biomechanical asymmetries became prominent from walking and running to turning activities. These findings underscore the different demands placed on children’s feet under varied movement conditions, highlighting how movement patterns shape foot biomechanics in dynamic activities. This has implications for understanding children’s adaptation to loads and the potential risks of asymmetric load distribution.

The analysis of COP trajectories revealed substantial left-right differences in stability strategies, particularly noticeable during stance phases across different activities. For walking, COP showed a tendency for pronation in the left foot and supination in the right, reflecting a relatively balanced load distribution during this low-impact activity. This alignment suggests that, in a steady gait, children’s feet exhibit minor asymmetries that still allow for balanced movement, likely due to the low speed and force requirements [29,30]. However, during running, COP trajectories diverged significantly between the left and right feet. The pronounced reliance on one foot to achieve stability at higher speeds reflects children’s adaptation to the demands of rapid adjustments during high-impact activities [31]. This finding aligns with prior studies indicating that increased speed and load in movement often heighten asymmetries, as children may unconsciously favor their dominant foot for stabilization and control [32,33,34].

In directional turning tasks, COP trajectory analysis revealed distinct directional shifts, with COP in the left foot leaning toward pronation for left turns and COP in the right foot moving toward supination for right turns. This trend was particularly pronounced in running turns, likely due to the complex balancing requirements and redirecting momentum at high speeds. Turning demands rapid adjustments of the center of mass and increased stability from the support foot [35,36], and the observed COP shifts reflect children’s adaptation to these demands. These findings indicate that children rely on specific foot regions to achieve stability during turns, especially during running. This reliance may reflect a developmental trend in which children’s neuromuscular systems still adapt to managing balance during quick, dynamic shifts [26,37,38]. Notably, this also raises considerations for injury prevention, as the increased load on the support foot during turns could lead to overuse or strain if the foot consistently bears these loads asymmetrically.

FBI findings further substantiated the observed COP asymmetries, demonstrating that balance control differs substantially between the left and right feet in straight and turning movements. In walking, the left foot tended slightly toward pronation and the right toward supination, suggesting balanced but minor asymmetry. However, the FBI asymmetry became more significant during running, especially in the mid-stance phase, indicating that the feet adopt distinct balance strategies to manage the increased demands of running. This more significant divergence in FBI suggests that running requires elevated foot stability and control, which may lead to an overreliance on one foot to maintain balance [39,40]. Previous studies similarly report that running placed significant stress on stability and control, which could amplify existing foot asymmetries as load increases [24,41]. This finding implies that high-demand movements may increase the risk of imbalance-related foot health issues, underscoring the need for targeted interventions to enhance bilateral stability during running.

The regional plantar pressure analysis highlighted areas where pressure distribution differed markedly between the left and right feet, with these differences intensifying with activity demands. For instance, pressure in the left second metatarsal region (M2) was lower during walking than in the right, suggesting relatively balanced load distribution in low-impact movement. However, during running, the first metatarsal (M1) on the left side showed significantly lower pressure than the right, while pressure was notably higher in the fourth (M4) and fifth metatarsal (M5) regions of the left foot. In turning tasks, particularly running turns, the support foot bore more significant localized pressures, with higher loads concentrated in the first and fifth metatarsal regions. This uneven pressure distribution suggests that children may rely more on specific areas of the support foot to stabilize during high-speed directional changes [26,40,42]. Such a pattern indicates that, as movement demands increase, certain foot regions experience heightened pressure, likely to compensate for rapid shifts in momentum and direction. This has critical implications for children’s foot health, as persistent asymmetric pressure distribution may predispose them to localized strain and injury [43,44].

These findings offer practical insights for supporting children’s foot health. Identifying asymmetries in foot biomechanics across varied activities can guide targeted interventions to promote more balanced gait patterns. For instance, balance training exercises could focus on reducing reliance on one foot, potentially mitigating injury risks linked to prolonged asymmetric load distribution. Additionally, the observed COP and FBI asymmetries may serve as early indicators of developing gait imbalances, which could aid in detecting potential issues in children’s foot development. Incorporating assessments of load distribution patterns into routine pediatric evaluations could allow for proactive management of foot health, particularly in children engaging in sports or other physically demanding activities.

This study has certain limitations. The relatively small sample size and focus on a specific age group may limit the generalizability of the findings. Further studies with more diverse samples, including gender, different age groups, and physical activity levels, would provide a more comprehensive understanding of these biomechanical patterns. Additionally, as the study was conducted in a controlled laboratory setting, future research could investigate these dynamics in real-world environments to improve the ecological validity of the results. Expanding research to consider footwear types and ground surfaces would offer deeper insights into how different conditions influence children’s foot biomechanics.

## 5. Conclusions

This study identified biomechanical differences between children’s left and right feet during walking, running, and turning tasks, showing that pressure is redistributed. Children may rely more heavily on either foot for stability and control, which became the dominant limb, as reflected in the center of pressure (COP), Foot Balance Index (FBI), and regional plantar pressure distributions. These findings highlight the importance of early identification and intervention for asymmetrical loading patterns to promote balance and reduce injury risk, providing valuable insights for future assessments and interventions in children’s gait and foot health.

## Data Availability

The data may be available upon reasonable request from the corresponding authors.
